# AMPK promotes TFEB transcriptional activity through dephosphorylation at both MTORC1-dependent and -independent sites

**DOI:** 10.1080/15548627.2026.2629720

**Published:** 2026-02-23

**Authors:** Florentina Negoita, Conchita Fraguas Bringas, Kristina Hellberg, Katarzyna M. Luda, Hongling Liu, Zhiyuan Li, Joyceline Cuenco, Jin-Feng Zhao, Gajanan Sathe, Ian G. Ganley, Gopal P. Sapkota, Kei Sakamoto

**Affiliations:** a Novo Nordisk Foundation Center for Basic Metabolic Research, University of Copenhagen, Copenhagen, Denmark; b Centre for Cardiovascular Science, Queen’s Medical Research Institute, University of Edinburgh, Edinburgh, UK; c Medical Research Council (MRC) Protein Phosphorylation and Ubiquitylation Unit, School of Life Sciences, University of Dundee, Dundee, UK

**Keywords:** BAY-3827, coordinated lysosomal expression and regulation, MK-8722, MTOR, reversible phosphorylation, TFE3

## Abstract

TFEB (transcription factor EB) is a critical regulator of lysosomal biogenesis, macroautophagy/autophagy and energy homeostasis through controlling expression of genes belonging to the coordinated lysosomal expression and regulation network. AMP-activated protein kinase (AMPK) has been reported to phosphorylate TFEB at three conserved C-terminal serine residues (S466, S467, S469) and these phosphorylation events were reported to be essential for transcriptional activation of TFEB. In sharp contrast to this proposition, we demonstrate that AMPK activation leads to the dephosphorylation of the C-terminal sites. We show that a synthetic peptide encompassing the C-terminal serine residues of TFEB is a poor substrate of AMPK in vitro. Treatment of cells with an AMPK activator (MK-8722), glucose deprivation or MTOR inhibitor (torin1) robustly dephosphorylated TFEB not only at the MTORC1-targeted N-terminal serine sites, but also at the C-terminal sites. Loss of function of AMPK abrogated MK-8722- but not torin1-induced dephosphorylation and induction of the TFEB target genes.

**Abbreviations:** AMPK: 5’-adenosine monophosphate-activated protein kinase; ACAC/ACC: acetyl-CoA carboxylase; AICAR: 5-aminoimidazole-4-carbox-amide ribonucleotide; CLEAR: coordinated lysosomal expression and regulation; DKO: double knockout; DMEM: Dulbecco’s modified Eagle’s medium; DMSO: dimethyl sulfoxide; DQ-BSA: self-quenched BODIPY® dye conjugates of bovine serum albumin; KI: knock-in; KO: knockout; MEFs: mouse embryonic fibroblasts; MTORC1: mechanistic target of rapamycin kinase complex 1; RRAGC: Ras related GTP binding C; RPTOR: regulatory associated protein of MTOR complex 1; RPS6KA/RSK: ribosomal protein S6 kinase A; RPS6KB1/S6K1: ribosomal protein S6 kinase B1; RT-qPCR: reverse transcription quantitative polymerase chain reaction; TFE3: transcription factor binding to IGHM enhancer 3; TFEB: transcription factor EB; ULK1: unc-51 like autophagy activating kinase 1; WT: wild-type

## Introduction

**Figure 1.**
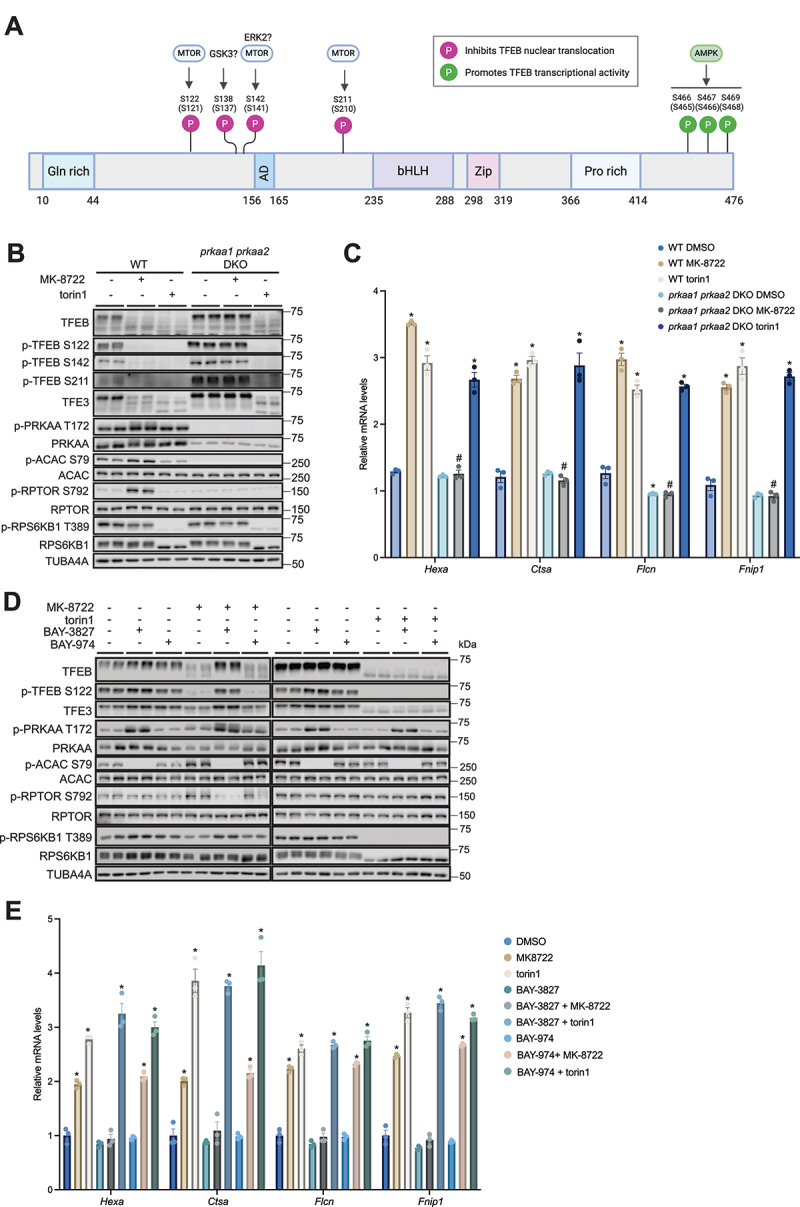
AMPK is dispensable for MTORC1-regulated (torin1-sensitive) transcriptional activity of TFEB. (A) Schematic domain structure and reported phosphorylation sites (validated by phospho-specific antibodies) of human TFEB. Mouse TFEB phosphorylation numbering in bracket. Illustration was created in BioRender (2026). https://biorender.com/ta3ks9q. (B and C) Wild type (WT) and *prkaa1 prkaa2* double knockout (DKO) mouse embryonic fibroblasts (MEFs) were treated with vehicle (0.1% DMSO), 10 μM MK-8722 or 100 nM torin1 for 6 h. The extracted proteins and RNA were subjected to immunoblot analysis with the indicated antibodies or RT-qPCR assay, respectively. Representative immunoblot images (B) and qPCR data (C; mean ± SEM) from three independent experiments are shown. Statistical analysis was performed using two-way ANOVA followed by Šidák’s multiple comparison test. *p < 0.05 vehicle *vs*. treatment; ^#^*p* < 0.05 DKO *vs*. WT within the indicated treatment. *n* = 2 per treatment condition for immnoblot and *n* = 3 per treatment condition for qPCR. (D and E) WT MEFs were pre-incubated with vehicle, 5 μM BAY-3827 or 5 μM BAY-974 for 30 min followed by treatment with 10 μM MK-8722 or 100 nM torin1 for 6 h. Representative immunoblot images (D) and qPCR data (E) from three independent experiments are shown. *n* = 2 per treatment condition for immnoblot and *n* = 3 per treatment condition for qPCR. (E) Data from one independent experiment with *n* = 3 replicates shown as mean ± SEM. A one-way ANOVA test with šídák’s multiple comparison was performed (*p < 0.05 vehicle *vs*. treatment).

TFEB (transcription factor EB) and related TFE3 (transcription factor binding to IGHM enhancer 3) are members of the basic helix-loop-helix leucine zipper family of transcription factors [1]. TFEB is a master regulator of lysosomal biogenesis, autophagy and cellular energy homeostasis through controlling expression of genes belonging to the coordinated lysosomal expression and regulation (CLEAR) network [2–4]. An important mechanism by which TFEB is regulated involves its shuttling between the surface of lysosomes, the cytoplasm, and the nucleus. Such dynamic changes in subcellular localization occur in response to nutrient fluctuations and various forms of cellular and energetic stresses, and are primarily mediated via changes in the phosphorylation of multiple conserved residues in TFEB [1,5] (Figure 1A). Under nutrient- and energy-repleted states, TFEB is held inactive in the cytosol by MTOR (mechanistic target of rapamycin) complex 1 (MTORC1) through phosphorylation of multiple serine residues (including S122, S142, S211) [1,6–9]. MTORC1 is recruited to the lysosomal membrane for activation by the RRAG GTPases, which are heterodimers composed of RRAGA or RRAGB bound to RRAGC or RRAGD. RRAGA/RRAGB-RRAGC/RRAGD complexes associate with the lysosomal membrane via binding to the pentameric lysosomal complex Ragulator [10,11].

TFEB phosphorylation by MTORC1 selectively requires a guanosine triphosphate (GTP)-activating protein (GAP) complex composed of the FLCN (folliculin) and FNIP1 (FLCN interacting protein 1) proteins [12]. The FLCN-FNIP1 complex promotes GDP loading of RRAGC [13–15], the inhibition which results in the detachment of TFEB and TFE3 from the lysosome and away from MTORC1 [12,16]. Upon inhibition of MTORC1 and/or activation of 5’-adenosine monophosphate-activated protein kinase (AMPK) in response to nutrient or energy depletion, TFEB undergoes dephosphorylation and nuclear translocation [6–9,17–19]. In the nucleus, TFEB directly binds to a specific E-box sequence (known as CLEAR motif) in the proximal promoters of numerous genes that regulate lysosomal and metabolic functions [2,3]. It has been reported that activated AMPK promotes nuclear translocation of TFEB through inhibition of MTORC1 via phosphorylation of RPTOR (regulatory associated protein of MTOR complex 1) and TSC2 (TSC complex subunit 2) [20], or via phosphorylation of FNIP1 and the resulting dissociation of RRAGC, MTORC1 and TFEB from the lysosome [4,17].

Notably, AMPK has been reported to phosphorylate TFEB at a cluster of three conserved C-terminal serine residues (S466, S467, S469) (Figure 1A), and these phosphorylation events are required for transcriptional activation of TFEB [19]. The study showed that in *prkaa*-null cells, while inhibition of MTORC1 by starvation or torin1 (a potent and selective ATP-competitive MTOR inhibitor) promoted TFEB localization to the nucleus, TFEB activity was abrogated. In addition, cellular introduction of a GFP-tagged TFEB mutant which carried mutations in the three C-terminal serine sites to alanine (S466A S467A S469A) did not disrupt the ability to promote nuclear translocation of TFEB, but abrogated transcriptional activity of TFEB upon activation of AMPK (with 5-aminoimidazole-4-carboxamide ribonucleotide [AICAR]) or inhibition of MTORC1 (with torin1) [19]. However, the mechanism by which AMPK-mediated phosphorylation regulates TFEB activity or whether phosphorylation of all three residues is required is unknown. Given that all three of these tightly clustered C-terminal serine residues of TFEB poorly match the AMPK substrate consensus motif [21,22], it is unlikely that AMPK phosphorylates all three sites (especially with the same stoichiometry). In the current study, we developed a series of phospho-site specific antibodies and synthetic peptides targeting or encompassing the C-terminal serine residues of TFEB and assessed the ability of AMPK to phosphorylate these sites in a cell-free assay, and studied phosphorylation and activity of TFEB in response to a highly-specific allosteric AMPK activator (MK-8722) and torin1 in cells genetically lacking AMPK or treated with a potent and highly-selective AMPK inhibitor (BAY-3827). In stark contrast to the previous study [19], here we demonstrate that AMPK activation promotes dephosphorylation of the C-terminal serine residues of TFEB in a similar manner as has been shown with other MTORC1-regulated N-terminal serine sites (S122, S142, S211) in cells. Moreover, we demonstrate that torin1-stimulated transcriptional activity of TFEB is highly preserved in *prkaa*-null cells.

## Results and discussion

### AMPK is dispensable for MTOR-regulated (torin1-sensitive) transcriptional activity of TFEB

Previous studies, including our own, utilized the widely-used AMP-mimetic pro-drug AICAR to pharmacologically activate AMPK in cells, and reported that AICAR robustly promoted dephosphorylation and nuclear localization of TFEB [18,19]. However, AICAR has been shown to produce numerous off-target effects through regulating for example AMP- and ZMP-sensitive enzymes [23–25]. We recently reported that AICAR treatment positively or negatively regulated (fold-change value of >1.3) a large proportion of differentially expressed genes [1026 out of 2053 in MEFs (mouse embryonic fibroblasts) and 754 out of 1718 in mouse primary hepatocytes, respectively] in an AMPK independent mechanism [18]. In contrast, a benzimidazole derivative compound 991 (also known as ex229), a precedent structural analog of MK-8722, which potently activates all AMPK trimeric complexes through binding in a pocket termed allosteric drug and metabolite (ADaM) site (located at the interface of the PRKAA/AMPKα catalytic subunit and PRKAB/AMPKβ regulatory subunit) [26,27] exhibited nearly exclusive specificity for targeting AMPK in both MEFs and hepatocytes [18]. Consistent with our previous data [28,29], we verified that MK-8722 (1 µM) potently activated AMPK and did not affect any other kinases (i.e., no >50% activation or inhibition) in a panel of 140 human protein kinases in a cell-free assay (Fig. S1A).

We treated wild-type (WT) and *prkaa1 prkaa2* double-knockout (DKO) MEFs with MK-8722 (10 µM) or torin1 (100 nM) for 2 or 6 h and assessed phosphorylation and transcriptional activity of TFEB by immunoblotting and reverse transcription quantitative PCR (RT-qPCR), respectively (Figure 1B,C, Figure S1B and C). Consistent with previous reports, a polypeptide reactive to the total PRKAA/AMPKα antibody was detectable in *prkaa1 prkaa2* DKO cells; however, this band likely represents a nonspecific signal, as no AMPK activity was detected following immunoprecipitation with the total PRKAA/AMPKα antibody [30]. In line with previous studies using 991 [17,18], MK-8722 treatment caused a robust increase in phosphorylation of PRKAA/AMPK and its *bona fide* substrates ACAC/ACC (acetyl-CoA carboxylase) and RPTOR in WT, but not in *prkaa1 prkaa2* DKO cells (Figure 1B, Figure S1B). Torin1 treatment abrogated phosphorylation of an MTOR substrate RPS6KB1/p70S6K1/S6K1 in WT and *prkaa1 prkaa2* DKO, while MK-8722 elicited only a marginal inhibitory effect on RPS6KB1 phosphorylation in WT (but not in *prkaa1 prkaa2* DKO) cells (Figure 1B, Figure S1B). Both MK-8722 and torin1 treatments caused profound dephosphorylation of TFEB at multiple established MTORC1-targeted residues (S122, S142, S211) [1] assessed by phospho-specific antibodies and electrophoretic mobility band shift of total TFEB (and also TFE3) in WT. We next measured previously validated transcripts of torin1- or AMPK activator-sensitive TFEB target genes (*Hexa*, *Ctsa*, *Flcn*, *Fnip1*) [3,18,19] by RT-qPCR (Figure 1C and Figure S1C). There was no difference in the expression of TFEB-targeted genes between vehicle-treated conditions in WT and *prkaa1 prkaa2* DKO, except a modest decrease in *Flcn*. Both MK-8722 and torin1 significantly increased (1.5–2-fold) transcripts of all four genes 2 and 6 h following the treatment in WT cells (Figure 1C, Figure S1C). While an MK-8722-stimulated increase in transcripts of TFEB target genes was abrogated in *prkaa1 prkaa2* DKO, in sharp contrast to the previous study [19], torin1 induced comparable increases in the target gene expression between WT and *prkaa1 prkaa2* DKO cells. To substantiate our findings using cells that genetically (i.e., constitutively) lacking AMPK, we also took a pharmacological approach and acutely inhibited AMPK using a highly selective and potent inhibitor (BAY-3827) [31–33]. PRKAA1 and PRKAA2 were among the kinases most potently inhibited by BAY-3827, and a cell-free selectivity screen against 331 kinases revealed that BAY-3827 had greater selectivity for AMPK [32] than the previously described AMPK and ULK1 inhibitor SBI-0206965 [34,35]. However, it should be noted that RPS6KA1/RSK1, RPS6KA2/RSK2, RPS6KA3/RSK3 and their related kinases (MSK1, MST3) were identified as off-targets of BAY-3827 [31,32]. Thus, a proper negative control BAY-974, which is structurally related to BAY-3827 but has no inhibitory activity on AMPK [31,32], was also employed. We observed that BAY-3827, but not BAY-974, abrogated MK-8722-stimulated phosphorylation of ACAC and RPTOR, as well as dephosphorylation of RPS6KB1, TFEB and TFE3 (Figure 1D). It should be noted that although BAY-3827 potently inhibits the catalytic activity of AMPK, it has been shown to increase PRKAA phosphorylation possibly due to its protective effect against Thr172 dephosphorylation [33]. Both BAY-3827 and BAY-974 treatment showed no detectable changes in torin1-sensitive/MTOR-regulated signaling or basal expression of the TFEB-target genes. BAY-3827 abrogated MK-8722-, but not torin1-stimulated induction of the TFEB-target gene expression (Figure 1E). In contrast, but as expected BAY-974 treatment did not affect MK-8722- or torin1-stimulated gene expression. Taken together, loss of AMPK function, whether through genetic or pharmacological means, did not affect torin1-stimulated gene expression of the selected TFEB targets.

### Torin1-regulated TFEB- and TFE3-dependent gene expression is profoundly preserved in cells lacking AMPK

**Figure 2.**
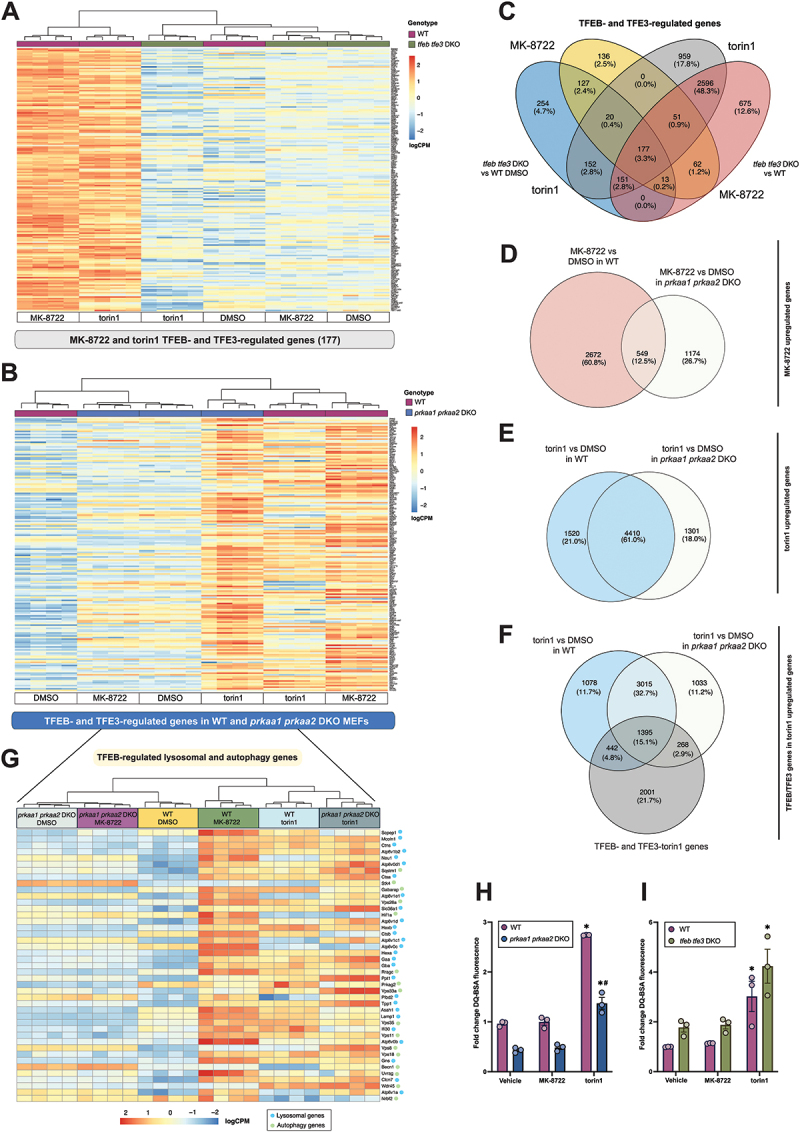
Unbiased messenger RNA sequencing substantiates dispensable role for AMPK in MTORC1-mediated/torin1-sensitive transcriptional activation of TFEB and TFE3. (A and B) Heatmaps of gene expression of MK-8722 and torin1-regulated genes in (A) WT and *tfeb tfe3* DKO MEFs and (B) in WT and *prkaa1 prkaa2* DKO MEFs. (C-F) venn diagrams depicting significantly upregulated genes across treatments, with (C) TFEB- and TFE3-regulated genes by MK-8722 and torin1 were obtained by selecting significantly downregulated genes in *tfeb tfe3* DKO MEF cells compared to DMSO WT and across treatment conditions. Significantly upregulated genes upon MK-8722 (D) and torin1 (E) treatment across WT and *prkaa1 prkaa2* DKO genotypes and (F) significantly upregulated TFEB- and TFE3-regulated genes present in torin1 upregulated genes. All significant genes were defined as having an absolute value (abs) iFold change (FC) ≥ 1.2 and false discovery rate (FDR) < 0.05. (G) heatmap of previously reported TFEB-regulated lysosomal and autophagy genes [3] present in current MK-8722- and torin1-regulated TFEB- and TFE3-dependent gene dataset. All expression data is shown as logCPM (log2 counts per million). (H, I) lysosomal proteolytic activity expressed as fold change in DQ-BSA fluorescence relative to WT vehicle (0.1% DMSO), in (H) *prkaa1 prkaa2* DKO MEFs or (I) *tfeb tfe3* DKO MEFs treated with 10 μM MK-8722 or 100 nM torin1 for 3 h. Representative data from three independent experiments are shown as mean ± SEM. Two-way ANOVA with šídák’s multiple comparison correction test was performed for statistical analysis (*p < 0.05 vehicle *vs*. treatment within the indicated genotype and # *p* < 0.05 WT *vs*. DKO within the indicated treatment).

TFEB plays a key role in maintaining cellular homeostasis through regulating the expression of genes belonging to the CLEAR network [2,3]. To unbiasedly assess the requirement of AMPK in the transcriptional activation of TFEB induced by pharmacological activation of AMPK or inhibition of MTOR, two separate transcriptome analyses were conducted involving WT and *tfeb tfe3* DKO MEFs and WT and *prkaa1 prkaa2* DKO MEFs (Fig. S2A-D). We initially sought to establish MK-8722- and torin1-sensitive TFEB- and TFE3-dependent genes in WT and *tfeb tfe3* DKO MEFs (Figure 2A,C, Fig. S2A and B). Bulk mRNA-Seq (messenger RNA-sequencing) revealed that 829 genes (15%) were commonly upregulated (FC (fold change) ≥ 1.2, false discovery rate (FDR) < 0.05) in response to torin1 and MK-8722 treatments compared to vehicle (DMSO) control in WT MEFs, with a group of genes that had downregulated expression in drug-treated *tfeb tfe3* DKO compared to WT MEFs (Fig. S2A). To study this gene subset, differential expression analysis was performed, and it identified genes induced by torin1 or MK-8722 that were also significantly downregulated (abs(FC ≥ 1.2), FDR < 0.05) in *tfeb tfe3* DKO compared to WT MEFs. These genes were then subjected to enrichment analysis, and their associated gene ontology functions were explored (Fig. S2E and F). This revealed that the downregulated genes in response to both torin1 and MK-8722 treatments in *tfeb tfe3* DKO compared to WT cells have cellular functions involved in autophagy, ion homeostasis, vacuolar transport and organization and lysosome organization and transport. As anticipated, some of the downregulated genes in torin1 *vs*. vehicle control in *tfeb tfe3* DKO compared to WT were found to be involved in regulatory functions of MTOR signaling (Fig. S2E). With MK-8722 treatment, endosomal transport functions were also enriched in downregulated *tfeb tfe3* DKO genes (Fig. S2F). Gene set enrichment analysis was performed on torin1- and MK-8722-treated cells compared to vehicle in *tfeb tfe3* DKO *vs*. WT to study the top biological pathway associated with TFEB and TFE3 functions in these treatments. This revealed that the highly enriched pathway for both MK-8722 and torin1 treatments was the lysosome (Fig. S2G and H). Furthermore, we observed that genes significantly downregulated (abs(FC ≥ 1.2), FDR < 0.05) in both torin1 and MK-8722 *tfeb tfe3* DKO MEFs were commonly related to lysosomal and autophagy functions, reinforcing the established roles for TFEB and TFE3 in regulating lysosomal processes downstream of MTOR and AMPK signaling pathways.

To identify torin1- and MK-8722-sensitive TFEB- and TFE3-dependent genes, we specifically focused on significantly downregulated genes in *tfeb tfe3* DKO compared to WT cells in these two treatments. The genes that fit this criterion and that were significantly downregulated in *tfeb tfe3* DKO compared to WT MEFs in both MK-8722 and torin1 treatments compared to vehicle were selected, and their expression profiles were visualized in a heatmap (Figure 2A). This identified a total of 177 commonly downregulated genes in torin1- and MK-8722-treated *tfeb tfe3* DKO compared to the treated WT MEFs (and from hereafter to be referred to as MK-8722- and torin1-sensitive TFEB- and TFE3-dependent genes).

We then determined whether the expression of TFEB- and TFE3-dependent genes was altered in *prkaa1 prkaa2* DKO MEFs compared to WT in response to MK-8722 and torin1. Differential expression analysis showed that 1007 (11.6%) and 807 genes (9.3%) were significantly upregulated (FC ≥ 1.2, FDR < 0.05) in response to torin1 and MK-8722, respectively, in WT MEFs compared to vehicle (Fig. S2C), and an unbiased heatmap was produced to visualize gene expression profiles (Fig. S2D). We then looked at the gene expression of TFEB- and TFE3-regulated genes in WT and *prkaa1 prkaa2* DKO sequencing data using intact ENSEMBL IDs to select matching genes and visualized their expression profile in a heatmap (Figure 2B). All TFEB- and TFE3-regulated genes were matched in the WT and *prkaa1 prkaa2* DKO dataset except for the pseudogene *Gm8615*.

As anticipated, the majority (87.5%) of MK-8722-stimulated genes in WT were not upregulated in *prkaa1 prkaa2* DKO cells, with only a 12.5% observed overlap (Figure 2D). In contrast, 61% of upregulated genes in response to torin1 treatment were conserved in both WT and *prkaa1 prkaa2* DKO (Figure 2E). Among those commonly upregulated genes, we observed that 15.1% (1395 genes) were predicted torin1-sensitive TFEB/TFE3-dependent genes (Figure 2F), including 43 genes (out of 63) that had been previously reported as direct TFEB target genes [3] (Figure 2G). The depicted gene expression profiles revealed that the majority of MK-8722-stimulated genes were highly expressed in WT, but not in *prkaa1 prkaa2* DKO cells, whereas torin1-stimulated genes were highly-to-moderately expressed in both genotypes. Collectively, unbiased transcriptome analysis revealed that AMPK is not essential for the induction of the vast majority of known TFEB- and TFE3-regulated genes in response to torin1.

### TFEB, TFE3 and AMPK are dispensable for torin1-stimulated lysosomal proteolytic activation

It has been reported that both AICAR and torin1 acutely (within 2 hours) increase lysosomal proteolytic activity in WT, but not in *prkaa1 prkaa2* DKO MEFs [19] as measured by microscopic image-based DQ-BSA fluorescence, a self-quenched albumin probe that fluoresces upon degradation. We performed quantitative cytometry-based DQ-BSA fluorescence analyses in WT, *prkaa1 prkaa2* DKO and *tfeb tfe3* DKO MEFs treated with vehicle, MK-8722 or torin1 for 3 h (Figure 2H and I). TFEB dephosphorylation, AMPK activation and MTOR inhibition (e.g., by monitoring changes in phosphorylation of downstream targets including ULK1 phosphorylation at S555 and S757 regulated by AMPK and MTOR, respectively) were confirmed by immunoblotting (**Fig. S2I and J**). Even though lysosomal proteolytic activity was reduced in *prkaa1 prkaa*2 DKO cells compared to WT in basal (vehicle-treated) state as reported [19], we observed relatively similar increase (2-fold) with torin1 in both genotypes (Figure 2H). Pharmacological activation of AMPK by MK-8722 had no effect on lysosomal proteolytic activity in both WT and *prkaa1 prkaa2* DKO cells. Notably, we observed a modestly higher basal activity in *tfeb tfe3* DKO compared to WT cells, and torin1, but not MK-8722, had comparable increase in lysosomal proteolytic activity between the genotypes (Figure 2I). This indicates that an MTOR-dependent, but TFEB- and TFE3-independent mechanism is responsible for torin1-induced lysosomal proteolytic activity in MEFs. Collectively, AMPK is required for basal proteolytic activity, but both AMPK and TFEB are dispensable for an acute torin1-stimulated lysosomal proteolytic activity in MEFs.

### AMPK activation and MTOR inhibition promotes TFEB dephosphorylation at C-terminal serine residues

A previous study using a cell-free assay showed that recombinant PRKAA1/AMPKα1-PRKAB1/β1-PRKAG1/γ1 complex phosphorylated purified TFEB protein and that AMPK-mediated phosphorylation of the purified GST-TFEB C-terminal fragment (415–476) was modestly or profoundly reduced in the fragment harboring single serine to alanine substitution (S467A or S469A) or triple substitutions (S466A S467A S469A), respectively [19]. Consequently, a polyclonal phospho-specific antibody against a triple-phosphorylated peptide (p-S466 p-S467 p-S469) was developed, and the study showed that AICAR treatment significantly increased immunoprecipitation of TFEB and TFE3 against the p-S466 S467 S469 antibody compared to vehicle control in WT, but not in *prkaa1 prkaa2* DKO cells [19]. However, the specificity of the p-S466 S467 S469 antibody was poorly documented, and whether the antibody had exclusively detected the triple-phosphorylated protein or cross-reacted with single and/or dual-phosphorylated or non-phosphorylated protein was not reported. Notably, basal phosphorylation of p-S466 S467 S469 was similar between WT and *prkaa1 prkaa2* DKO cells [19], indicating that these sites can also be phosphorylated by other kinases independently of AMPK.

**Figure 3.**
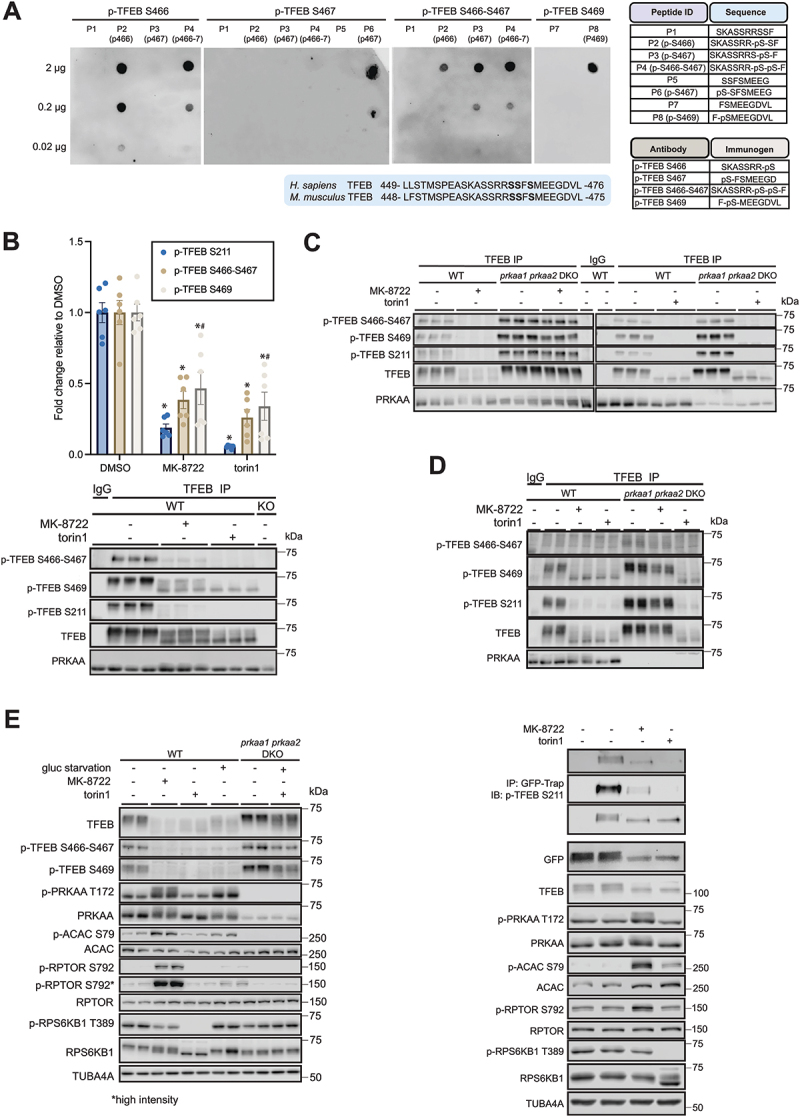
AMPK activation induces dephosphorylation of C-terminal phospho-sites of TFEB. (A) amino acid sequence alignment of human and mouse TFEB proteins encompassing C-terminal serine residues. Amino acid sequence of immunogen used for generation of phospho-specific antibodies. Sequence of peptides used for dot blot analysis. The lowercase letter “p” preceding a single-letter amino acid abbreviation signifies that the amino acid is phosphorylated. Images of dot blot assay for the p-TFEB-S466, -S467, -S466 S467 (dual) and -S469 antibodies. (B and C) WT MEFs alone (B) or both WT and *prkaa1 prkaa2* DKO MEFs (C) were treated with vehicle (0.1% DMSO), 10 μM MK-8722 or 100 nM torin1 for 1 h. Lysates from *tfeb tfe3* DKO (KO) MEFs were used as negative control for detection of total and phospho-TFEB (B). TFEB was immunoprecipitated (IP) from protein lysates and the immunoprecipitants were blotted with the indicated antibodies. IgG was used as negative control. Shown are representative immunoblot images and quantification of signals from *n* = 2 separate experiments containing *n* = 3 replicates per condition shown as mean ± SEM. A two-way ANOVA test using Tukey test to correct for multiple comparisons was conducted, with *p < 0.01 (DMSO *vs*. treatments) and ^#^*p* < 0.05 (S211*vs*. S469). (D) WT and *prkaa1 prkaa2* DKO HEK293 cells were treated with vehicle (0.1% DMSO), 10 μM MK-8722 or 100 nM torin1 for 1 h. TFEB was immunoprecipitated from protein lysates and the immunoprecipitants were blotted with the indicated antibodies. IgG was used as negative control. Representative immunoblot images from two independent experiments are shown. (E) WT and *prkaa1 prkaa2* DKO MEFs were glucose-deprived (in glucose-free medium) for 1 h. Treatment with vehicle (0.1% DMSO), 10 μM MK-8722 or 100 nM torin1 for 1 h was used as control. Representative immunoblot images from three independent experiments are shown. *n* = 2 per treatment condition. (F) TFEB-GFP was immunoprecipitated using GFP-Trap from lysates of TFEB-GFP knock-in (KI) MEFs treated for 1 h with vehicle (0.1% DMSO), 10 μM MK-8722 or 100 nM torin1. Immunoblot analysis was performed using the indicated antibodies. Representative immunoblot images from three independent experiments are shown.

We developed individual single (in human TFEB p-S466, p-S467, p-S469 and in mouse TFEB p-S465, p-S466, p-S468) and dual p-S466 S467 (p-S465 S466 in mouse) TFEB antibodies and initially performed dot blot analyses to assess specificity and sensitivity of the respective antibodies (Figure 3A). To avoid confusion over the TFEB residue numbering, particularly for the C-terminal serine residues, between human and mouse, we hereafter refer exclusively to the human TFEB numbering. To maximize the likelihood of generating site-specific antibodies against single p-S466 and p-S467 antibodies, we designed the antigen peptides with p-S466 and p-S467 positioned at the N-terminal or C-terminal ends, respectively, ensuring that phosphorylation at one site does not interfere with the other (Figure 3A). The p-S466 antibody recognized both singly phosphorylated p-S466 and dually phosphorylated p-S466 S467 peptides, while the p-S467 antibody specifically detected singly phosphorylated p-S467 peptide. The p-S466 S467 antibody recognized both dually phosphorylated p-S466 and p-S467 and singly phosphorylated p-S467 peptides with similar efficacy but cross-reacted to a lesser extent with singly phosphorylated p-S466 peptide. The p-S469 antibody specifically detected the p-S469 peptide, although with lower sensitivity compared to other phospho-specific antibodies tested (Figure 3A).

We assessed the phosphorylation of C-terminal serine residues of TFEB in response to MK-8722 (10 µM) or torin1 (100 nM) for 1 h in MEFs. TFEB dephosphorylation, AMPK activation and MTOR inhibition in response to the treatment were confirmed in lysates by immunoblotting (Fig. S3A and B). We immunoprecipitated TFEB using a total TFEB antibody (or IgG as negative control) and blotted with the p-TFEB antibodies. *tfeb tfe3* DKO MEF lysates were used as additional negative controls. Consistent with our earlier findings (Figure 1B), both MK-8722 and torin1 caused robust dephosphorylation of TFEB S211 (Figure 3B,C). In sharp contrast to the previous study [19], we observed that AMPK activation by MK-8722 and MTOR inhibition by torin1, resulted in a robust dephosphorylation (40–60%) of TFEB C-terminal serine residues (Figure 3B). We confirmed that MK-8722-mediated, but not torin1-mediated, dephosphorylation of TFEB C-terminal serine residues is AMPK dependent (Figure 3C) similar to the regulation of other known MTORC1 sites assessed earlier (Figure 1B) or in previous studies [17,18]. These findings were further replicated in HEK293 cells (Figure 3D). In addition to pharmacological activation, we stimulated AMPK through glucose starvation, a more physiologically relevant condition. As expected, glucose starvation caused a comparable increase in AMPK and ACAC/ACC1 phosphorylation in WT MEFs to that observed with MK-8722 treatment, but to a much lesser extent in RPTOR phosphorylation (Figure 3E). Glucose starvation also promoted dephosphorylation of TFEB C-terminal serine residues in WT, an effect that was largely diminished in *prkaa1 prkaa2* DKO MEFs (Figure 3E).

To complement the antibody-based approach, we performed mass spectrometry-based analysis of TFEB C-terminal serine phosphorylation. We generated C-terminally GFP-tagged TFEB knock-in (TFEB-GFP KI) MEFs, treated them with vehicle, MK-8722, or torin1 for 1 h, and immunoprecipitated TFEB-GFP protein using GFP-trap beads (Figure 3F, S3C and D). The immunoprecipitants were subjected to quantitative mass spectrometry analysis (Fig. S3E-H). We found that S467, relative to total TFEB, was consistently dephosphorylated by ~50% in response to both MK-8722 and torin1 (Fig. S3H). This observation was further confirmed by immunoblotting TFEB-GFP immunoprecipitants following GFP-trap affinity isolation (Figure 3F). Collectively, these results demonstrate that pharmacological activation of AMPK or inhibition of MTOR in cells results in a robust dephosphorylation of TFEB not only at the well-characterized MTORC1-dependent N-terminal serine sites (S122, S142, S211), but also MTORC1-independent C-terminal serine sites.

Although a previous study reported that recombinant AMPK could phosphorylate the C-terminal serine residues of TFEB in a cell-free assay, this may represent an in vitro artifact. To quantitatively assess phosphorylation of the C-terminal serine residues in TFEB that serve as AMPK substrates, relative to benchmarked/optimized peptide substrates (such as SAMS peptide, derived from the 13-residue sequence surrounding Ser79 of ACAC/ACC1, with a serine-to-alanine substitution of at position 5) [36,37], we designed synthetic peptides encompassing the C-terminal serine residues of TFEB. In addition, derivative peptides containing single or dual serine to alanine substitution were generated. To facilitate peptide binding to phosphocellulose paper, three arginine residues were appended to the C-terminus (Fig. S3I). In the absence of MK-8722, robust activity was observed toward AMARA and SAMS peptides (Fig. S3I), while only negligible activity was detected against TFEB C-terminal peptides, whether WT or serine-to-alanine-substituted. Even though MK-8722 modestly enhanced activity against TFEB C-terminal peptides, it was ~10–30 fold less compared to that against SAMS and AMARA peptides (Fig. S3I). Taken together, these results suggest that the C-terminal peptide of TFEB containing three serine residues (S466, S467 and S469) is a poor substrate of AMPK, which is consistent with previous studies and bioinformatics motif prediction that all three of C-terminal serine residues of TFEB and TFE3 poorly match the AMPK substrate consensus motif [21,22].

It remains unknown whether phosphorylation of C-terminal serine sites in TFEB is directly or indirectly regulated by MTORC1 [38] or other kinases. Although AKT was shown to phosphorylate S467 on TFEB in a cell-free assay [39], this has not been validated in cellular or tissue contexts, for example using an antibody- or mass spectrometry-based approaches. We stimulated WT MEFs with IGF1, which stimulates AKT and MTOR, in the presence or absence of MK-8722 and assessed TFEB phosphorylation. IGF1 treatment alone resulted in a modest increase (40–50%) in TFEB phosphorylation on S142 (an MTORC1 site), but not S466 S467, whereas neither IGF1 alone nor IGF1 in combination with MK-8722 altered the phosphorylation of S142 and S466 S467 (Fig. S3J). This suggests that either the AKT-MTOR signaling axis is not required or its activation alone is insufficient to promote phosphorylation of the S466 and S467 sites.

Recently, either activated AMPK via the allosteric activator 991 (an analog of MK-8722) or electron transport chain inhibitors, was shown to phosphorylate FNIP1, resulting in inhibition of FLCN-FNIP1 GAP activity [17]. This inhibition promotes accumulation of RRAGC in its GTP-bound form, leading to dissociation of RRAGC, MTORC1, and TFEB from the lysosome, and consequently reducing TFEB phosphorylation (as evidenced by gel mobility shift) and enhancing nuclear translocation. To further explore this mechanism, we assessed TFEB phosphorylation at multiple sites using phospho-specific antibodies following transient expression of FLAG-tagged WT, a GTP-locked mutant RRAGC^Q120L^, or a GDP-locked mutant RRAGC^S75N^ in HEK293 cells treated with vehicle or MK-8722. The GDP-locked RRAGC mutant consistently showed lower expression levels than WT or GTP-locked RRAGC mutant (Fig. S3K). Consistent with previous findings [17], the GDP-locked RRAGC expression markedly increased phosphorylation of TFEB at S122, and this phosphorylation was resistant to MK-8722-induced dephosphorylation compared to WT or GTP-locked RRAGC (Fig. S3K). A similar pattern was observed at the C-terminal serine phosphorylation site (S469) (Fig. S3K and L). In line with these findings and with previous studies [4,17,40], torin1 significantly increased TFEB – lysosome association, whereas AMPK activation by MK-8722, either alone or in combination with torin1, substantially reduced this association (Fig. S3M).

Responsible kinase(s) and phosphatase(s) that regulate reversible phosphorylation of TFEB C-terminal serine sites (S466, S467, S469) remain unknown. In addition to AKT and MTOR [38,39], protein kinase C (PKC) [41] has been also proposed to phosphorylate S466, S467, 469 in cell-free assays, and using a motif-based profile scanning approach (SCANSITE 4.0: https://scansite4.mit.edu/) protein kinase A (PKA) and aurora kinase are predicated as potential kinases phosphorylating S467. PPP3/calcineurin and PPP2/PP2A have been shown to be phosphatases for TFEB (e.g., MTORC1 sites) [42,43] and it would be interesting to determine if they dephosphorylate C-terminal sites under basal and also AMPK activator- or torin1-treated conditions in cells.

### Effect of alanine mutation of serine residue(s) in the C-terminal serine-rich motif on phosphorylation of other neighboring serine site(s)

Finally, we examined if loss or reduced phosphorylation at specific sites within the C-terminal serine-rich motif of TFEB influences phosphorylation of neighboring serine residues. To this end FLAG-TFEB WT and mutants (substitution of serine to alanine or threonine) were ectopically expressed in HEK293 cells, and cell lysates were analyzed by immunoblotting with antibodies against phosphorylated TFEB C-terminal serine residues (Fig. S3N). Consistent with the dot blot analysis (Figure 3A), we observed detection of p-S466 S467 signal in WT but not in cells expressing single S466A, S467A, double S466A S467A or S466T S467T mutant. We further observed that S467A, but not S466A, showed a profound downward band shift. Notably, S469A, but not S469T, markedly increased p-S466 S467 signal. Single S466A, S467A and double S466A S467A reduced detection of p-S469 signal. Since both S466 and S467 residues are not included in the immunogen peptide (Figure 3A), their alanine mutation should not have affected recognition by the p-S469 antibody. These results further validated the specificity of the p-TFEB antibodies and suggested a potential hierarchical phosphorylation mechanism. However, the interpretation of mutagenesis experiments should be approached with caution, as artificial mutations can introduce structural alterations. To date, no high-resolution crystal or cryo-EM structures have resolved the C-terminal serine cluster of TFEB, which is classified as an intrinsically disordered region [44]. Previous work has shown that introducing a triple TFEB S466A S467A S469A mutant into WT cells blocks MTORC1-regulated TFEB activity [19]. However, it remains unclear if this effect reflects the loss of phosphorylation itself or an artefactual conformational change induced by the alanine substitutions.

In conclusion, we demonstrate that AMPK activation and MTORC1 inhibition leads to dephosphorylation of conserved C-terminal serine sites in TFEB, while AMPK is dispensable for torin1-sensitive transcriptional TFEB activation. Future investigations are warranted to understand mechanisms underlying the reversible phosphorylation of the C-terminal serine sites and their functional significance.

## Materials and methods

### Cell lines, culture and treatment

WT and *prkaa1 prkaa2* DKO MEFs [30,45] were provided by Benoit Viollet (Institut Cochin INSERM U1016). WT and *tfeb tfe3* DKO MEFs [46] were provided by Rosa Puertollano (National Institutes of Health). HEK293 cells were purchased from Invitrogen (R75007). *Prkaa1 prkaa2* DKO HEK293 cells were provided by D. Grahame Hardie (University of Dundee). TFEB-GFP KI mice were generated by Taconic Biosciences through a constitutive KI of EGFP in the murine *Tfeb* gene via Easi-CRISPR-Cas9-mediated gene editing. The sequence for the open reading frame of EGFP was inserted between the last amino acid and the translation termination codon in exon 8. Spontaneously immortalized MEFs were generated by Core Facility for Transgenic Mice (Department of Experimental Medicine, University of Copenhagen) according to the previously published protocol [47]. Cells were cultured in DMEM (Dulbecco’s Modified Eagle Medium [Invitrogen, 31,966]) supplemented with 10% (v:v) fetal bovine serum (Sigma, F7524) and 1% (v:v) penicillin-streptomycin (Substrate and Sterile Center, University of Copenhagen) in a 37°C incubator with 5% CO_2_. For glucose-starvation experiments, cells were cultured in DMEM without glucose (Invitrogen, A14430) supplemented with 10% (v:v) fetal bovine serum (Sigma, F7524) and pyruvate, glutamine, 1% (v:v) penicillin-streptomycin (all from Substrate and Sterile Center, University of Copenhagen). All cells were regularly tested and confirmed negative for the presence of mycoplasma throughout the experimental period. The following reagents were used for cell treatment: DMSO (Sigma, D4540), MK-8722 (Glixx Laboratories Inc, GLXC-11445 and MedChemExpress, HY-111363), torin1 (Selleck Chemicals, S2827), BAY-3827 (MedChemExpress, HY-112083), BAY-974 (provided by Structural Genomics Consortium Frankfurt, Germany) and IGF1 (Thermo Fisher, PHG0071).

### Transient transfection

HEK293 cells at 80% confluence were transiently transfected with the plasmids encoding FLAG-tagged *TFEB* or *RRAGC* using TransIT-X2 Dynamic Delivery System (Mirus, MIR6000). The next day after plating the cells in 6-well plates, the cells in one well were incubated with TransIT-X2:DNA complexes containing 3.5 µl TransIT-X2 reagent and 900 ng plasmid DNA in 250 µl Opti-MEM medium (Gibco, 51,985–026). The cells were left for 18 h and then medium was replaced with full growth medium (DMEM supplemented with 10% FBS and 1% penicillin-streptomycin) for another 24 h followed by cell lysis. Following the treatment with the indicated drugs or vehicle, the cells were washed with room temperature PBS (Substrate and Sterile Center, University of Copenhagen) and lysed in lysis buffer (50 mM Tris-HCl [Sigma, T1503], pH 7.5, 1 mM ethylenediaminetetraacetic acid disodium salt dihydrate [EDTA; Sigma, E5134], 1 mM ethylene glycol-bis(2-aminoethylether)- *N,N,N′,N′*-tetraacetic acid [EGTA; Sigma, E3889], 270 mM sucrose [Sigma, S7903], 1% [w:v] Triton X-100 [Sigma, X100], 20 mM glycerol-2-phosphate disodium [Sigma, G9422], 50 mM sodium fluoride [NaF; Sigma, 201,154], 5 mM sodium pyrophosphate decahydrate [Sigma, 221,368], 1 mM DL-dithiothreitol [DTT; Sigma, 43,815] containing protease and phosphatase inhibitors (500 uM phenylmethylsulfonyl fluoride [PMSF; Sigma, P7626], 1 mM benzamidine hydrochloride [Sigma, 199,001], 1 μg/ml leupeptin [Sigma, L2884], 1 μg/ml pepstatin A [Sigma, P5318], 1 μM microcystin-LR [Enzo Life Sciences, ALX-350–012], 1 mM sodium orthovanadate [Sigma, S6508]). The lysates were clarified by centrifugation at 6000 x *g* for 10 min at 4°C and total protein concentration was determined using Bradford reagent (ThermoFisher Scientific, 23,200) and BSA as standard. The lysates were snap frozen in liquid nitrogen and stored at −80°C till further analysis. The following plasmids were obtained from MRC PPU Reagents and Services (University of Dundee): pCMV5 TFEB 3xFLAG (identifier: DU67838), pCMV5 TFEB S466A 3xFLAG (identifier: DU73282), pCMV5 TFEB S467A 3xFLAG (identifier: DU73283), pCMV5 TFEB S466A S467A 3xFLAG (identifier: DU7328), pCMV5 TFEB S469A 3xFLAG (identifier: DU73878), pCMV5 TFEB S466T S467T 3xFLAG (identifier: DU80259), pCMV5 TFEB S469T 3xFLAG (identifier: DU80257). The *RRAGC* plasmids were obtained from European Plasmid Repository: pcDNA3-FLAG-hRRAGC (identifier: EPR Plasmid #5), pcDNA3-FLAG-hRRAGC S75N (low-nucleotide) (identifier: EPR Plasmid #7), pcDNA3-FLAG-hRRAGC Q120L (GTP) (identifier: EPR Plasmid #9).

### Western blotting

Proteins in the lysates were denatured at 100°C for 5 min in Laemmli sample buffer (50 mM Tris, pH 6.8, 2% sodium dodecyl sulfate [SDS; Sigma, 74,255], 0.5 mM EDTA (Sigma, E5134), 10% glycerol [Sigma, G5516], 0.01% bromophenol blue [Sigma, 114,391]) and stored at −20°C until analysis. The denatured proteins were separated by SDS-PAGE on Mini-PROTEAN Tetra Cell (Bio-Rad Laboratories, Denmark) on 7–8% handcast mini polyacrylamide gels (acrylamide [Bio-Rad, 1,610,146], *N,N,N*′,*N*′-tetramethylethylenediamine (TEMED [Sigma, T9281]), ammonium persulfate [Sigma, 248,614], sodium dodecyl sulfate (SDS [Sigma, 74,255]). The separated proteins were transferred from the polyacrylamide gel to the nitrocellulose membrane (Sigma, GE10600002) by Mini Trans-Blot Cell system (Bio-Rad Laboratories, Denmark). Membranes with proteins were blocked with 3% (w:v) skim milk (Sigma, 70,166) in Tris-buffered saline (TBS; 20 mM Tris, 136 mM NaCl) containing 0.1% (w:v) Tween 20 (Sigma, P7949) (TBST). After blocking, the membranes with proteins were incubated with primary antibodies diluted in 4% bovine serum albumin (BSA [Sigma, A7030]) in TBST containing 0.02% sodium azide (Sigma, 71,290), overnight at 4°C. After washing with TBST, horseradish peroxidase (HRP)-conjugated secondary antibodies diluted in 3% skim milk in TBST were added to the membrane and incubated for 45 min at room temperature. The membranes were washed with TBST. The protein-antibody complexes on the membranes were visualized by Odyssey XF Imager (LI-COR Biotech, LLC, Germany) after incubation of the membrane with the enhanced chemiluminescence substrate (Millipore, WBKLS0500), with an integration time of 0.5 min for most of the blots. For some proteins, a higher integration time was required, such as 2 min or 5 min.

The primary antibodies used were: ACAC/ACC (Cell Signaling Technology, 3676; 1:1,000), p-ACAC/ACC S79 (Cell Signaling Technology, 3661; 1:1,000), RPTOR/raptor (Cell Signaling Technology, 2280; 1:1,000), p-RPTOR/raptor S792 (Cell Signaling Technology, 2083; 1:1,000), PRKAA/AMPKα (Cell Signaling Technology, 2532; 1:1,000), p-PRKAA/AMPKα T172 (Cell Signaling Technology, 2535; 1:1,000), RPS6KB1/p70S6K (Cell Signaling Technology, 2708; 1:1,000), p-RPS6KB1/p70S6K T389 (Cell Signaling Technology, 9234; 1:1,000), TFEB (Bethyl Laboratories, A303-673A; 1:1,000), TFEB (Cell Signaling Technology, 4240; 1:1,000), p-TFEB S122 (Cell Signaling Technology, 86,843; 1:1,000), p-TFEB S211 (Cell Signaling Technology, 37,681; 1:1,000), p-TFEB S142 (Millipore, ABE1971; 1:1,000), TFE3 (Cell Signaling Technology, 14,779 and 81,744; 1:1,000), p-ULK1 S555 (Cell Signaling Technology, 5869; 1:1,000), p-ULK1 S757 (Cell Signaling Technology, 14,202; 1:1,000), ULK1 (Cell Signaling Technology, 8054; 1:1,000), AKT (Cell Signaling Technology, 4691; 1:1,000), p-AKT S473 (Cell Signaling Technology, 4060; 1:1,000), TUBA4A/tubulin (Sigma, T6074; 1:5,000), VCL/vinculin (Cell Signaling Technology, 13901T; 1:1000), GFP (Chromotek, 3H9, 1:1000), FLAG (Sigma, F1804; 1:1000). Affinity-purified p-TFEB S466 (immunogen sequence: SKASSRR-*p*S, where *p* denoted phosphorylated residue), p-TFEB S467 (immunogen sequence: *p*S-FSMEEGD), p-TFEB S466 S467 (immunogen sequence SKASSRR-*p*S-*p*S-F), p-TFEB S469 (immunogen sequence: F-*p*S-MEEGDVL) and p-TFE3 S321 (immunogen sequence: KAITVSN-*p*S-CPAELPN) were generated by Yenzym Antibodies, LLC and used at 1 µg/ml dilution. The secondary antibodies used were: HRP-conjugated anti-rabbit (Jackson ImmunoResearch Europe Ltd, 111–035-144; 1:10,000), HRP-conjugated anti-rat (Jackson ImmunoResearch Europe Ltd, 112–035-143; 1:10,000), HRP-conjugated anti-mouse (Bio-Rad, 1,706,516; 1:10,000).

### Dot blot

The phospho- and non-phospho-peptides encompassing the clustered C-terminal serine residues of TFEB/TFE3 were synthesized by GL Biochem (Shanghai) Ltd and had the following sequences: P1 (SKASSRSSF), P2 (SKASSR-*p*S-SF, where *p* denoted phosphorylated residue), P3 (sequence SKASSRS-*p*S-F), P4 (SKASSR-*p*S-*p*S-F), P5 (SSFSMEEG), P6 (*p*S-SFSMEEG), P7 (FSMEEGDVL), P8 (F-*p*SMEEGDVL). Increasing doses of peptides were spotted onto a nitrocellulose membrane and allowed to air-dry for 30 min. The membranes were further blocked with 3% skim milk in TBST and the rest of the steps followed the protocol mentioned above in Western blotting.

### Immunoprecipitation

Protein lysates (0.5–1 mg) were incubated with 20 µl 50% slurry Protein G Sepharose (Cytiva, 17,061,802) coupled with the relevant antibodies on a VXR basic Vibrax orbital shaker (IKA, Denmark) for 16 h at 4°C. At the end of the incubation time, Sepharose beads containing the immunoprecipitated protein were washed three times with lysis buffer supplemented with 500 mM NaCl (Sigma, S9888). The proteins captured on the beads were eluted from the beads by adding Laemmli buffer for 10 min at 65°C with vibration (1000 rpm). The eluents were subjected to SDS-PAGE as described above. The antibodies used for immunoprecipitation were: TFEB (Proteintech, 13,372–1-AP; 2 µg antibody for 0.5 mg lysate collected from MEFs), TFEB (Abcam, ab267351; 1 µg antibody for 1 mg lysate collected from HEK293 cells) and negative control IgG from the same species (Cell Signaling Technology, 2729). For detection of protein-antibody complex, a light-chain specific secondary antibody (Jackson ImmunoResearch Europe Ltd, 211–032-171; 1:10,000) was used to avoid the potential interference with the heavy chain of the antibody.

### In vitro AMPK activity assay

AMPK activity assay was performed as previously described [48]. Recombinant human PRKAA1-PRKAB1-PRKAG1 trimeric complex (SignalChem Biotech, P47-110GH) was diluted in enzyme dilution buffer (50 mM Tris, pH 7.4, 0.1 mM EGTA, 1 mg/ml BSA, 1 mM DTT). The kinase reaction was performed in buffer A (50 mM HEPES, pH 7.4, 0.1 mM EGTA, 10 mM MgCl_2_, 150 mM NaCl, 1 mM DTT) containing 100 μM ATP (Sigma, A2383), 200 μM peptide and [γ-^33^P]ATP (Hartmann Analytic, SCF-301–12) at 30°C for 10 min. The enzyme reaction was stopped by spotting 75% of the reaction mixture onto cation-exchange phosphocellulose paper (obtained from SVI Phosphocellulose (https://www.svi.edu.au/resources/phosphocellulose_paper/)) and the phosphocellulose paper was washed in 75 mM phosphoric acid (Sigma, W290017), and then in acetone (Sigma, 179,124). After washing, the radioactivity of the ^33^P incorporated into the substrate peptides was detected by Cerenkov counting in a Hidex 600SLe scintillation counter (Hidex Oy, Finland) for 5 min. The substrate peptides used were: AMARA (AMARAASAAALARRR, MRC PPU Reagents and Services) and SAMS (HMRSAMSGLHLVKRR, MRC PPU Reagents and Services). Peptides based on the C-terminal sequence of TFEB, TFEB C-terminal WT (ASSRRSSFSMEEGRRR), S466A (ASSRRASFSMEEGRRR), S467A (ASSRRSAFSMEEGRRR), S466A-S467A (ASSRRAAFSMEEGRRR) and S469A (ASSRRSSFAMEEGRRR) were obtained from GL Biochem (Shanghai) Ltd.

### RNA sequencing and bioinformatic analysis

Total RNA was isolated using QIAwave RNeasy kit (QIAGEN, 74,536) with on-column DNAse treatment using Qiagen RNase-Free DNase set (QIAGEN, 79,256) following manufacturer’s protocol. Messenger RNA sequencing was performed by the Single-Cell Omics platform at the Novo Nordisk Foundation Center for Basic Metabolic Research. Libraries were prepared using the Universal Plus mRNA-seq protocol (Tecan Life Sciences, 0520) as recommended by the manufacturer. Libraries were quantified with Qubit fluorometer (ThermoFisher Scientific, Waltham, USA), quality checked using a TapeStation instrument (Agilent Technologies, Waldbronn, Germany) and subjected to 52-bp paired-end sequencing on a NovaSeq 6000 (Illumina, San Diego, USA).

Data were processed using nf-core/rnaseq v3.11.2 of the nf-core collection of workflows [49]. The pipeline was executed with Nextflow v23.04.1 [50] with the reference genome set as *Mus musculus* mm10 GRCm38 release 102.Workflow parameters included STAR aligner and unique molecular identifiers (UMI) were considered using “–with_umi – skip_umi_extract – umitools_umi_separator ‘’”: parameters. Differential expression analysis was carried out in RStudio version 4.2.0 using the package edgeR [51] and a batch-effect removal was performed using the package limma [52]. Venn diagrams were produced using the R package ggvenn (https://github.com/yanlinlin82/ggvenn), with significant upregulated or downregulated genes described as abs(FC ≥ 1.2) and FDR < 0.05. Heatmap figures of gene expression in log2 counts per million (logCPM) were produced with the R package pheatmap [53]. Gene ontology analysis was conducted using clusterProfiler [54] showing biological process (BP) ontology and a p-adjust method set to “BH” (Benjamini & Hochberg), with significant genes described as abs(FC ≥ 1.2) and FDR < 0.05. Gene set enrichment analysis (GSEA) was performed using gseaplot R package [55].

### qPCR

At indicated time points, cells were washed once with PBS (Substrate and Sterile Center, University of Copenhagen) and lysed with Buffer RLT (QIAGEN, 79,216) supplemented with 2-mercaptoethanol (Sigma, M6250; 1:100 dilution). RNA was isolated, and on-column DNase treated with RNAse-Free DNase (QIAGEN, 79,256), using RNEasy mini kit (QIAGEN, 74,106) or QiaWave (QIAGEN, 74,536) according to the manufacturer’s instructions. RNA concentration and quality was analyzed by a NanoDrop One Spectrophotometer (ThermoFisher Scientific, Wilmington, USA). 2 μg RNA was reverse transcribed using Superscript III cDNA synthesis kit (Invitrogen, 18,080), dNTPs (Invitrogen, 10,297–018), RNaseOUT (Invitrogen, 10,777,019), random primers (Roche, 11,034,731,001) and nuclease-free water (Ambion, AM9937) in a 20-μl reaction as per the manufacturer’s instructions. cDNA was diluted 20x and 2 μl was used for qPCR reactions (10 μl total) using indicated primers (Table S1) and PrecisionPLUS qPCR Master Mix with SYBRgreen (Primer Design, Z_PPLUS-SY-20 ml) on a LightCycler480 (Roche Diagnostics A/S, Denmark). For each experiment, cDNA was diluted to generate a standard curve to analyze primer efficiency. The delta delta Ct method was used to calculate gene expression and data was normalized to mean of *Hprt* and *Tbp*.

### DQ-BSA assay

The cells were seeded on 12-well plates and the following day were loaded with 10 μg/ml DQ Red BSA (Invitrogen, D12051) in complete medium for 2 h at 37°C. At the end of the loading period, medium with DQ Red BSA was removed, and the cells were washed once with PBS and then treated with MK-8722 or with torin1 for 3 h. Cells were washed with PBS once and detached with trypsin (Gibco, 25,300–054). Trypsinized cells were spun down at 500 x *g* for 5 min and cell pellets were resuspended for 20 min in 2% (v:v) formaldehyde (Sigma, 252,549) solution in PBS for fixation at room temperature. Subsequently, cells were washed with PBS, spun down at 600 x *g* for 5 min and the resultant cell pellets were resuspended in PBS for analysis. Analytical flow cytometry was performed on CytoFLEX S cytometer (Beckman Coulter ApS, Denmark). Cell debris was gated out from total cells, and single cell gate was placed to analyze mean fluorescence intensity of the DQ-BSA dye by excitation with the yellow/green laser (561 nm) and detection in the channel with a band pass filter 610/20 nm. Data analysis was performed using FlowJo software (Tree Star).

### Immunoprecipitation followed by mass spectrometry

TFEB-GFP KI MEF cells were first lysed in NP-40 lysis buffer (50 mM HEPES, pH 7.4, 150 mM NaCl, 1 mM EDTA, 10% glycerol, 1% NP-40 [Thermo Fisher Scientific, 85,125], 1 mM DTT, 1 mM PMSF [Sigma, 93,482], 1.15 mM sodium molybdate [Sigma, 243,655], 4 mM sodium tartrate [Sigma, 71,995], 10 mM β-glycerophosphate [Sigma, G9422], 1 mM sodium fluoride, 1 mM sodium orthovanadate [Sigma, 567,540] and 1x cOmplete^TM^ EDTA-free protease inhibitor cocktail [Roche, 11,873,580,001]). Clarified lysates (60 mg protein) were incubated with GFP-Trap Agarose beads (30 μl packed beads; ChromoTek, gta) for 4 h on a rotating wheel at 4°C. Following incubation, beads were washed 3x with standard lysis buffer. Bead-bound proteins were denatured and eluted in 2x LDS for 5 min at 95°C. Samples were then filtered through Spin-X columns (Costar, 8161) to remove the beads from the eluate. The filtered eluate was loaded onto a 4–12% Bis-Tris gradient gel (Invitrogen, NW04120BOX) and proteins were separated by SDS-PAGE. Gels were stained with InstantBlue (Abcam, ab119211) and subsequently de-stained in deionized water. A small portion of the eluate was retained for analysis and validation by western blotting. To minimize potential protein contaminants, all steps from this point were performed under a laminar flow hood. Disposable scalpels were used to cut protein bands of interest from the InstantBlue-stained gels into 1–2 cm cubes, which were subsequently transferred into LoBind 1.5 ml microcentrifuge tubes (Eppendorf, 0030108116). Gel pieces were washed once in HPLC grade water, and then shrunk in anhydrous acetonitrile (ACN) for 5 min with gentle shaking. The ACN was aspirated, and gel pieces were re-swollen with 50 mM Tris-HCl, pH 8.0 for 5 min with shaking. The shrinking-swelling process was repeated once more, and the proteins within the gel pieces were reduced with 5 mM DTT in 50 mM Tris-HCl, pH 8.0 for 20 min at 65°C. Next, the proteins within the gel pieces were alkylated with 20 mM IAA (iodoacetamide) in 50 mM Tris-HCl, pH 8.0 for 20 min at room temperature in the dark. Gel pieces were then shrunk again in ACN for 5 min, dried and re-swollen in 50 μl of 50 mM TEAB (triethylammonium bicarbonate; Sigma, T7408), pH 8.0 buffer containing 5 mg/ml trypsin (Promega, V5111). After removing excess trypsin, gel pieces were covered in 50 mM TEAB and samples incubated in a shaker overnight at 37°C for tryptic digestion. An equivalent volume of ACN was added to the digest for 15 min with shaking and the supernatant was collected into a fresh LoBind 1.5 ml microcentrifuge tube. Gel pieces were then re-swollen with 0.1% (v:v) trifluoroacetic acid (TFA) for 5 min with shaking, and peptides were extracted twice with ACN for 5 min each with shaking. After each extraction, the supernatant was collected and combined with the previous supernatants. The supernatants were then dried by vacuum centrifugation using a SpeedVac.

### Mass spectrometry analysis

The total run time was set to 70 min. The mass spectrometer was operated in a data-dependent acquisition mode. A survey full scan MS (from m/z 350 to 1200) was acquired in the Orbitrap at a resolution of 60,000 (at 200 m/z). The automatic gain control (AGC) target for MS1 was set as 1.2 × 106, and ion filling time was set as 50 ms. The most abundant ions with charge state ≥2 were isolated in a 3-s cycle, fragmented by using high-energy collision dissociation fragmentation with 30% normalized collision energy, and detected at a mass resolution of 15,000 at 200 m/z. The AGC target for MS/MS was set as standard and ion filling time was set at custom, whereas dynamic exclusion was set to 30 s with a 10-ppm (parts per million) mass window. These DDA data were searched using MaxQuant software. Protein sequence from UniProt was used as a FASTA sequence to search the data. Trypsin was set as proteases with a maximum of two missed cleavages were allowed. Oxidation of Met, deamidation of Asn and Gln and phosphorylation of Ser, Thr and Tyr were set as a variable modification and carbamidomethylation of Cys was set as a fixed modification. The default instrument parameters for MS1 and MS2 tolerance were used, and the data was filtered for 1% PSM, peptide and Protein level FDR.

### Immunofluorescence

HEK293 cells were seeded on coverslips 24 h prior to treatment with DMSO, torin1 (250 nM), MK-8722 (10 µM), or a combination of torin1 and MK-8722 for 3 h. Cells were fixed with 3.7% paraformaldehyde, permeabilized with 0.3% Triton X-100, and blocked with 1% BSA. Immunostaining was performed using anti-LAMTOR1 (Cell Signaling Technology, 8975) and anti-TFEB (Cell Signaling Technology, 91767) primary antibodies at 4°C overnight, followed by fluorescent secondary antibodies for 1 h at room temperature. Nuclei were counterstained with Hoechst, and samples were mounted using ProLong Glass Antifade. Images were acquired using a Zeiss LSM880 confocal microscope with a 63× objective and analyzed using CellProfiler [56] to quantify TFEB translocation to LAMTOR1 positive structures and nuclear TFEB intensity.

### Statistical analysis

All experiments were independently performed at least 2–3 times with duplicates or triplicates for each experimental condition. The results are showed as mean ± SEM (standard error of the mean). Statistical significance was evaluated by a one-way or two-way ANOVA as indicated in the figure legends. Statistical significance was accepted at */#*p* < 0.05.To correct for multiple comparisons, Šídák’s test or Tukey’s test was performed as indicated in the figure legends. All statistical testing was performed in GraphPad Prism version 10.1.0.

## Supplementary Material

Edited_Supplementary_Figures_final_version_final.docx

## Data Availability

All RNA sequencing data pertaining to this project has been deposited in the Gene Expression Omnibus (GEO) repository and is available under accession number GSE270826 as part of a SuperSeries, where data from WT and *tfeb tfe3* DKO MEF cells is denoted as X1 (GSE270824) and WT and *prkaa1 prkaa2* DKO MEF cell data as X2 (GSE270825). https://www.ncbi.nlm.nih.gov/geo/query/acc.cgi?acc=GSE270826. The raw mass spectrometry data related to this project has been deposited to the ProteomeXchange Consortium via the PRIDE partner repository under the accession code PXD055410.

## References

[1] Puertollano R, Ferguson SM, Brugarolas J, et al. The complex relationship between TFEB transcription factor phosphorylation and subcellular localization. Embo J. 2018;37(11). doi: 10.15252/embj.201798804 PMC5983138 29764979

[2] Sardiello M, Palmieri M, di Ronza A, et al. A gene network regulating lysosomal biogenesis and function. Science. 2009;325(5939):473–477. doi: 10.1126/science.1174447 19556463

[3] Palmieri M, Impey S, Kang H, et al. Characterization of the CLEAR network reveals an integrated control of cellular clearance pathways. Hum Mol Genet. 2011;20(19):3852–3866. doi: 10.1093/hmg/ddr306 21752829

[4] Settembre C, Zoncu R, Medina DL, et al. A lysosome-to-nucleus signalling mechanism senses and regulates the lysosome via mTOR and TFEB. Embo J. 2012;31(5):1095–1108. doi: 10.1038/emboj.2012.32 22343943 PMC3298007

[5] Takla M, Keshri S, Rubinsztein DC. The post-translational regulation of transcription factor EB (TFEB) in health and disease. EMBO Rep. 2023;24(11):e57574. doi: 10.15252/embr.202357574 37728021 PMC10626434

[6] Martina JA, Chen Y, Gucek M, et al. Mtorc1 functions as a transcriptional regulator of autophagy by preventing nuclear transport of TFEB. Autophagy. 2012;8(6):903–914. doi: 10.4161/auto.19653 22576015 PMC3427256

[7] Roczniak-Ferguson A, Petit CS, Froehlich F, et al. The transcription factor TFEB links MTORC1 signaling to transcriptional control of lysosome homeostasis. Sci Signal. 2012;5(228):ra42. doi: 10.1126/scisignal.2002790 22692423 PMC3437338

[8] Vega-Rubin-de-Celis S, Pena-Llopis S, Konda M, et al. Multistep regulation of TFEB by MTORC1. Autophagy. 2017;13(3):464–472. doi: 10.1080/15548627.2016.1271514 28055300 PMC5361595

[9] Napolitano G, Esposito A, Choi H, et al. mTOR-dependent phosphorylation controls TFEB nuclear export. Nat Commun. 2018;9(1):3312. doi: 10.1038/s41467-018-05862-6 30120233 PMC6098152

[10] Sancak Y, Bar-Peled L, Zoncu R, et al. Ragulator-Rag complex targets mTORC1 to the lysosomal surface and is necessary for its activation by amino acids. Cell. 2010;141(2):290–303. doi: 10.1016/j.cell.2010.02.024 20381137 PMC3024592

[11] Sancak Y, Peterson TR, Shaul YD, et al. The Rag GTPases bind raptor and mediate amino acid signaling to mTORC1. Science. 2008;320(5882):1496–1501. doi: 10.1126/science.1157535 18497260 PMC2475333

[12] Napolitano G, Di Malta C, Esposito A, et al. A substrate-specific mTORC1 pathway underlies Birt-Hogg-Dube syndrome. Nature. 2020;585(7826):597–602. doi: 10.1038/s41586-020-2444-0 32612235 PMC7610377

[13] Tsun ZY, Bar-Peled L, Chantranupong L, et al. The folliculin tumor suppressor is a GAP for the RRAGC/D GTPases that signal amino acid levels to MTORC1. Mol Cell. 2013;52(4):495–505. 24095279 10.1016/j.molcel.2013.09.016 PMC3867817

[14] Shen K, Rogala KB, Chou HT, et al. Cryo-em structure of the human FLCN-FNIP2-Rag-Ragulator complex. Cell. 2019;179(6):1319–1329 e8. doi: 10.1016/j.cell.2019.10.036 31704029 PMC7008705

[15] Lawrence RE, Fromm SA, Fu Y, et al. Structural mechanism of a Rag GTPase activation checkpoint by the lysosomal folliculin complex. Science. 2019;366(6468):971–977. doi: 10.1126/science.aax0364 31672913 PMC6945816

[16] Li K, Wada S, Gosis BS, et al. Folliculin promotes substrate-selective MTORC1 activity by activating RRAGC to recruit TFE3. PLOS Biol. 2022;20(3):e3001594. doi: 10.1371/journal.pbio.3001594 35358174 PMC9004751

[17] Malik N, Ferreira BI, Hollstein PE, et al. Induction of lysosomal and mitochondrial biogenesis by AMPK phosphorylation of FNIP1. Science. 2023;380(6642):eabj5559. doi: 10.1126/science.abj5559 37079666 PMC10794112

[18] Collodet C, Foretz M, Deak M, et al. Ampk promotes induction of the tumor suppressor Flcn through activation of Tfeb independently of mTOR. Faseb J. 2019;33(11):12374–12391. doi: 10.1096/fj.201900841R 31404503 PMC6902666

[19] Paquette M, El-Houjeiri L, Cz L, et al. Ampk-dependent phosphorylation is required for transcriptional activation of TFEB and TFE3. Autophagy. 2021;17(12):3957–3975. doi: 10.1080/15548627.2021.1898748 33734022 PMC8726606

[20] Young NP, Kamireddy A, Van Nostrand JL. Ampk governs lineage specification through TFEB-dependent regulation of lysosomes. Genes Dev. 2016;30(5):535–552. doi: 10.1101/gad.274142.115 26944679 PMC4782048

[21] Gwinn DM, Shackelford DB, Egan DF, et al. AMPK phosphorylation of raptor mediates a metabolic checkpoint. Mol Cell. 2008;30(2):214–226. doi: 10.1016/j.molcel.2008.03.003 18439900 PMC2674027

[22] Hardie DG, Schaffer BE, Brunet A. Ampk: an energy-sensing pathway with multiple inputs and outputs. Trends Cell Biol. 2016;26(3):190–201. doi: 10.1016/j.tcb.2015.10.013 26616193 PMC5881568

[23] Guigas B, Sakamoto K, Taleux N, et al. Beyond AICA riboside: in search of new specific AMP-activated protein kinase activators. IUBMB Life. 2009;61(1):18–26. doi: 10.1002/iub.135 18798311 PMC2845387

[24] Hunter RW, Hughey CC, Lantier L, et al. Metformin reduces liver glucose production by inhibition of fructose-1–6-bisphosphatase. Nat Med. 2018;24(9):1395–1406. doi: 10.1038/s41591-018-0159-7 30150719 PMC6207338

[25] Steinberg GR, Carling D. Amp-activated protein kinase: the current landscape for drug development. Nat Rev Drug Discov. 2019;18(7):527–551. doi: 10.1038/s41573-019-0019-2 30867601

[26] Xiao B, Sanders MJ, Carmena D, et al. Structural basis of AMPK regulation by small molecule activators. Nat Commun. 2013;4:3017. doi: 10.1038/ncomms4017 24352254 PMC3905731

[27] Langendorf CG, Kemp BE. Choreography of AMPK activation. Cell Res. 2015;25(1):5–6. doi: 10.1038/cr.2014.163 25475061 PMC4650591

[28] Bultot L, Jensen TE, Lai YC, et al. Benzimidazole derivative small-molecule 991 enhances AMPK activity and glucose uptake induced by AICAR or contraction in skeletal muscle. Am J Physiol Endocrinol Metab. 2016;311(4):E706–E719. doi: 10.1152/ajpendo.00237.2016 27577855 PMC5241553

[29] Myers RW, Guan HP, Ehrhart J, et al. Systemic pan-AMPK activator MK-8722 improves glucose homeostasis but induces cardiac hypertrophy. Science. 2017;357(6350):507–511. doi: 10.1126/science.aah5582 28705990

[30] Goransson O, McBride A, Hawley SA, et al. Mechanism of action of A-769662, a valuable tool for activation of AMP-activated protein kinase. J Biol Chem. 2007;282(45):32549–32560. doi: 10.1074/jbc.M706536200 17855357 PMC2156105

[31] Lemos C, Schulze VK, Baumgart SJ, et al. The potent AMPK inhibitor BAY-3827 shows strong efficacy in androgen-dependent prostate cancer models. Cellular Oncol. 2021;44(3):581–594. doi: 10.1007/s13402-020-00584-8 PMC12980684 33492659

[32] Fraguas Bringas C, Ahangar MS, Cuenco J, et al. Mechanism and cellular actions of the potent AMPK inhibitor BAY-3827. Sci Adv. 2025;11(34):eadx2434. doi: 10.1126/sciadv.adx2434 40845097 PMC12372887

[33] Hawley SA, Russell FM, Ross FA, et al. Bay-3827 and Sbi-0206965: potent AMPK inhibitors that paradoxically increase Thr172 phosphorylation. Int J Mol Sci. 2023;25(1):453. doi: 10.3390/ijms25010453 38203624 PMC10778976

[34] Dite TA, Langendorf CG, Hoque A, et al. Amp-activated protein kinase selectively inhibited by the type II inhibitor SBI-0206965. J Biol Chem. 2018;293(23):8874–8885. doi: 10.1074/jbc.RA118.003547 29695504 PMC5995511

[35] Ahwazi D, Neopane K, Markby GR, et al. Investigation of the specificity and mechanism of action of the ULK1/AMPK inhibitor SBI-0206965. Biochem J. 2021;478(15):2977–2997. doi: 10.1042/BCJ20210284 34259310 PMC8370752

[36] Davies SP, Carling D, Hardie DG. Tissue distribution of the AMP-activated protein kinase, and lack of activation by cyclic-AMP-dependent protein kinase, studied using a specific and sensitive peptide assay. Eur J Biochem. 1989;186(1–2):123–128. doi: 10.1111/j.1432-1033.1989.tb15185.x 2574667

[37] Weekes J, Ball KL, Caudwell FB, et al. Specificity determinants for the AMP-activated protein kinase and its plant homologue analysed using synthetic peptides. FEBS Lett. 1993;334(3):335–339. doi: 10.1016/0014-5793(93)80706-Z 7902296

[38] Pena-Llopis S, Vega-Rubin-de-Celis S, Schwartz JC, et al. Regulation of TFEB and V-ATPases by mTORC1. Embo J. 2011;30(16):3242–3258. doi: 10.1038/emboj.2011.257 21804531 PMC3160667

[39] Palmieri M, Pal R, Nelvagal HR, et al. Mtorc1-independent TFEB activation via AKT inhibition promotes cellular clearance in neurodegenerative storage diseases. Nat Commun. 2017;8:14338. doi: 10.1038/ncomms14338 28165011 PMC5303831

[40] Martina JA, Puertollano R. Rag GTPases mediate amino acid-dependent recruitment of TFEB and MITF to lysosomes. J Cell Biol. 2013;200(4):475–491. 23401004 10.1083/jcb.201209135 PMC3575543

[41] Ferron M, Settembre C, Shimazu J, et al. A RANKL-PKCbeta-TFEB signaling cascade is necessary for lysosomal biogenesis in osteoclasts. Genes Dev. 2013;27(8):955–969. 23599343 10.1101/gad.213827.113 PMC3650231

[42] Medina DL, Di Paola S, Peluso I, et al. Lysosomal calcium signalling regulates autophagy through calcineurin and TFEB. Nat Cell Biol. 2015;17(3):288–299. doi: 10.1038/ncb3114 25720963 PMC4801004

[43] Martina JA, Puertollano R. Protein phosphatase 2A stimulates activation of TFEB and TFE3 transcription factors in response to oxidative stress. J Biol Chem. 2018;293(32):12525–12534. doi: 10.1074/jbc.RA118.003471 29945972 PMC6093222

[44] Tesei G, Trolle AI, Jonsson N, et al. Conformational ensembles of the human intrinsically disordered proteome. Nature;626(8000):897–904. doi: 10.1038/s41586-023-07004-5 38297118

[45] Laderoute KR, Amin K, Calaoagan JM, et al. 5’-amp-activated protein kinase (AMPK) is induced by low-oxygen and glucose deprivation conditions found in solid-tumor microenvironments. Mol Cell Biol. 2006;26(14):5336–5347. 16809770 10.1128/MCB.00166-06 PMC1592699

[46] Martina JA, Diab HI, Brady OA, et al. Tfeb and Tfe3 are novel components of the integrated stress response. Embo J. 2016;35(5):479–495. doi: 10.15252/embj.201593428 26813791 PMC4772850

[47] Xu J. Preparation, culture, and immortalization of mouse embryonic fibroblasts. Curr Protoc Mol Biol. 2005;Chapter 28:Unit 28 1. 70(1). doi: 10.1002/0471142727.mb2801s70 18265366

[48] Hunter RW, Foretz M, Bultot L, et al. Mechanism of action of compound-13: an alpha1-selective small molecule activator of AMPK. Chem Biol. 2014;21(7):866–879. 25036776 10.1016/j.chembiol.2014.05.014 PMC4104029

[49] Ewels PA, Peltzer A, Fillinger S, et al. The nf-core framework for community-curated bioinformatics pipelines. Nat Biotechnol. 2020;38(3):276–278. doi: 10.1038/s41587-020-0439-x 32055031

[50] Di Tommaso P, Chatzou M, Floden EW, et al. Nextflow enables reproducible computational workflows. Nat Biotechnol. 2017;35(4):316–319. doi: 10.1038/nbt.3820 28398311

[51] Robinson MD, Dj M, Gk S. EdgeR: a Bioconductor package for differential expression analysis of digital gene expression data. Bioinformatics. 2010;26(1):139–140. 19910308 10.1093/bioinformatics/btp616 PMC2796818

[52] Ritchie ME, Phipson B, Wu D, et al. Limma powers differential expression analyses for RNA-sequencing and microarray studies. Nucleic Acids Res. 2015;43(7):e47. doi: 10.1093/nar/gkv007 25605792 PMC4402510

[53] Kolde R. Pheatmap: pretty heatmaps. R package version 1.0.12. 2018.

[54] Wu T, Hu E, Xu S, et al. Clusterprofiler 4.0: a universal enrichment tool for interpreting omics data. Innov (Camb). 2021;2(3):100141. doi: 10.1016/j.xinn.2021.100141 PMC8454663 34557778

[55] Innis SE, Reinaltt K, Civelek M, et al. Gseaplot: a package for customizing gene set enrichment analysis in R. J Comput Biol. 2021;28(6):629–631. doi: 10.1089/cmb.2020.0426 33861629 PMC8219183

[56] Stirling DR, Swain-Bowden MJ, Lucas AM, et al. CellProfiler 4: improvements in speed, utility and usability. BMC Bioinf. 2021;22(1):433. doi: 10.1186/s12859-021-04344-9 PMC8431850 34507520

