# Supplementary material for: AMPK promotes TFEB transcriptional activity through dephosphorylation at both MTORC1-dependent and -independent sites
Source: Autophagy. 2026 Feb 23;22(6):1386–400. doi: 10.1080/15548627.2026.2629720 (PMC13185439; doi:10.1080/15548627.2026.2629720)
Supplement: Edited_Supplementary_Figures_final_version_final.docx [file KAUP_A_2629720_SM0623.docx]

**Table S1**. Primer sequences used for this study.

| Gene | Forward 5-3 | Reverse 5-3 |
| --- | --- | --- |
| *Tbp* | CCTTGTACCCTTCACCAATGAC | ACAGCCAAGATTCACGGTAGA |
| *Hprt* | GCCCCAAAATGGTTAAGGTT | CAAGGGCATATCCAACAACA |
| *Hexa* | GCTGAGGGCACGTTCTTTATC | GCGAGATGTATCCAGCAGTACG |
| *Ctsa* | GAGCAGAACGACAACTCCCT | TGCCCACAATTCGAGACACT |
| *Flcn* | TGGATCGGATCTACCTCATCA | TGGACATCCAAACTGCTCTG |
| *Fnip1* | GATGCGTGTTCATGTCAAGG | GGAGAGTGGGTGCTTGCTAC |

**Figure S1.**Kinase selectivity profile for MK-8722 and AMPK-dependent and MK8722-induced TFEB dephosphorylation and activation. (**A**) A screen of 140 human protein kinases (n = 2 per kinase, with or without 1 μM MK-8722) was performed *in vitro* using the MRC-PPU Premier Screen service (<https://www.kinase-screen.mrc.ac.uk/services/premier-screen>). Results are expressed as mean ± SEM. The PRKAA1-PRKAAB2-PRKAG1 (AMPK) complex (positive control) is shown in blue. (**B and C**) WT and *prkaa1 prkaa2* DKO MEFs were treated with vehicle (0.1% DMSO), 10 μM MK-8722 or 100 nM torin1 for 2 h followed by cell lysis for protein and RNA extraction. (**B**) Protein extracts were subjected to immunoblot analysis using the indicated antibodies. Representative immunoblot images from three independent experiments are shown. (**C**) mRNA levels of the indicated genes were detected by RT-qPCR. Data from one experiment is shown. n=3 per treatment condition. A two-way ANOVA with Šídák’s multiple comparison was performed (* *p* < 0.05 vehicle *vs.* treatment and # *p* < 0.05 WT *vs.* DKO).

**Figure S2.** Unbiased mRNA sequencing in wild type (WT) and double-knockout (DKO) *tfeb tfe3* or *prkaa*1 *prkaa2* MEFs (mouse embryonic fibroblasts) treated with MK-8722 or torin1 reveals drug-regulated TFEB and TFE3 genes are associated with lysosomal functions.  (**A**) Heatmap showing the gene expression profile of WT and *tfeb tfe3* DKO MEFs treated with DMSO (0.1%), 100 nM torin1 or 10 μM MK-8722 shown in logCPM (log2 counts per million). (**B**) Venn diagram representing significant upregulated genes (FC ≥ 1.2 and FDR < 0.05) across torin1- and MK-8722-treated WT and *tfeb tfe3* DKO cells compared to vehicle (DMSO). (**C**) Venn diagram of significant upregulated genes (FC ≥ 1.2 and FDR < 0.05) of torin1 and MK-8722 treatments *vs.* vehicle in WT and *prkaa1 prkaa2* DKO MEFs. (**D**) Unbiased heatmap showing the gene expression profile in logCPM (log2 counts per million) of WT and *prkaa1 prkaa2* DKO MEFs treated with DMSO, torin1 or MK-8722. (**E**) Gene ontology enrichment analysis of the significant downregulated genes in *tfeb tfe3* DKO compared to WT in torin1 *vs.* DMSO and (**F**) MK-8722 *vs.* DMSO conditions, with the top 20 biological processes (BP) categories shown alongside gene count and FDR values. (**G**) GSEA (Gene set enrichment analysis) was performed on DMSO *vs.* MK-8722 and (**H**) DMSO *vs.* torin1 in *tfeb tfe3* DKO *vs.* WT conditions, examining the *tfeb tfe3* DKO effect compared to WT, with the running enrichment score for the top pathway in both contrasts shown accordingly. (**I and J**) WT, *prkaa1 prkaa2* DKO (**I**) or *tfeb tfe3* DKO (**J**) MEFs were treated with vehicle (0.1% DMSO), 10 μM MK-8722 or 100 nM torin1 for 1 h and immunoblot analysis was performed as controls for the experiments presented in Fig. 2H and 2I. Representative immunoblot images from three independent experiments are shown.

**Figure S3.**AMPK activation induces dephosphorylation of C-terminal Ser residues of TFEB. (**A and B**) WT, *tfeb tfe3* DKO (A) or *prkaa1 prkaa2* DKO (B) MEF cells were treated with vehicle (0.1% DMSO), 10 μM MK-8722 or 100 nM torin1 for 1 h and immunoblot analysis was performed as controls for the experiments presented in Fig. 3B and 3C. Representative immunoblot images from three independent experiments are shown. (**C and D**) Detection of GFP by immunoblotting and Coomassie blue gel staining in samples used for mass spectrometry analysis. Immunopurified TFEB-GFP from TFEB-GFP KI MEF cells treated with vehicle (0.1% DMSO) “D”, 10 μM MK-8722 “M” or 100 nM torin1 “T” for 1 h was subjected to immunoblot analysis using anti-GFP antibody. (**E-G**) Extracted Ion Chromatogram (XIC) Analysis of the RSSFSMEEGDVL phospho-peptide. (**E**) XIC extracted from raw data for the precursor RSSFSMEEGDVL phospho-peptide under different treatment conditions as follows: DMSO (vehicle), 10 μM MK-8722 and 100 nM torin1. (**F**) Bar plots for comparative XIC peak area extracted from raw data in (E) for the precursor RSSFSMEEGDVL phospho-peptide across DMSO, MK-8722, or torin1 treatment. (**G**) MS/MS spectra confirming the identity of the RSpSFSMEEGDVL phospho-peptide, showing fragment ion coverage and phospho-site localization. (**H**) GFP-trap affinity-isolated samples were analyzed by quantitative mass spectrometry. Phospho-peptide corresponds to TFEB (S467) quantification based on intensity in the DMSO, MK-8722 and torin1 samples using data-dependent analysis. TFEB protein quantification based on LFQ (label-free quantification) in the DMSO, MK-8722 and torin1 samples using data-dependent analysis. TFEB phosphorylation in DMSO, MK-8722 and torin1 treated samples were quantified by taking ratio of the TFEB-S467 phosphorylation intensity with total TFEB quantification. (**I**) In vitro AMPK activity assay using different peptide substrates, including SAMS, AMARA and peptides corresponding to WT or mutated C-terminal serine residues of TFEB. Shown as mean ± SEM is n=3 per condition from n=2 separate experiments. Statistical analysis was performed using a 2-way ANOVA test using Tukey’s test for multiple comparisons correction. *p* values were accepted as < 0.05 where **p* (recombinant [rec]AMPK vs recAMPK + MK-8722), #*p* (recAMPK + MK-8722 AMARA peptide vs recAMPK + MK-8722 across indicated peptides) and †*p* (recAMPK + MK-8722 SAMS peptide vs recAMPK + MK-8722 across indicated peptides). (**J**) WT MEF cells were serum-starved for 16 h. MEFs were incubated with vehicle or MK-8722 (10 μM) for 30 min followed by an addition of IGF-1 (100 ng/ml) or vehicle for 30 min. Immunoblot analysis was performed using the indicated antibodies. Representative images from two independent experiments are shown. (**K and L**) HEK293 cells were transiently transfected with FLAG-tagged RRAGC WT, RRAGC S75N “RRAGC GDP” or RRAGC Q120L “RRAGC GTP” and treated with vehicle or 10 μM MK-8722 for 1 h. (**K**) Cell lysates were subjected to immunoblot analysis using the indicated antibodies. Representative immunoblot images from three independent experiments are shown. (**L**) TFEB was immunoprecipitated from protein lysates and the immunoprecipitants were blotted with the indicated antibodies and IgG was used as negative control. Representative immunoblot images from two independent experiments are shown. (**M**) HEK293 cells were treated with torin1 (250 nM), MK-8722 (10 µM), or both for 3 h, then fixed and stained for LAMTOR1 (red), TFEB (green), and Hoechst (blue) and imaged by confocal microscopy. Boxed section is shown magnified to right of image. Dashed line represents position of nuclear envelope and arrowheads highlight examples of TFEB-LAMTOR1 colocalization. Scale bar: 10 µm. Signal quantification of microscopy data shown above panel, with data from three independent experiments where **p* < 0.05 vehicle *vs.* treatments and ^#^*p* < 0.05 torin1 condition vs MK-8722 and torin1 + MK-8722. Statistical analysis was performed using one-way ANOVA followed by Tukey’s multiple comparisons correction test. (**N**) HEK293 cells were transiently transfected with WT and different serine to alanine or threonine mutants of TFEB. Immunoblot analysis was performed using the indicated antibodies. Representative images from one experiment.
